# Increase of nerve growth factor levels in the human herniated intervertebral disc: can annular rupture trigger discogenic back pain?

**DOI:** 10.1186/ar4674

**Published:** 2014-07-28

**Authors:** Yasuchika Aoki, Arata Nakajima, Seiji Ohtori, Hiroshi Takahashi, Fusako Watanabe, Masato Sonobe, Fumiaki Terajima, Masahiko Saito, Kazuhisa Takahashi, Tomoaki Toyone, Atsuya Watanabe, Takayuki Nakajima, Makoto Takazawa, Koichi Nakagawa

**Affiliations:** Department of Orthopaedic Surgery, Toho University Sakura Medical Center, 564-1 Shimoshizu, Sakura, Chiba, 285-8741 Japan; Department of Orthopaedic Surgery, Graduate School of Medicine, Chiba University, 1-8-1 Inohana, Chuo-ku, Chiba-city, Chiba, 260-8677 Japan; Department of Orthopaedic Surgery, Teikyo University Chiba Medical Center, 3426-3 Anesaki, Ichihara-city, Chiba, 299-0111 Japan; Department of Orthopaedic Surgery, Eastern Chiba Medical Center, 3-6-2 Okayamadai, Togane, Chiba, 283-8686 Japan

## Abstract

**Introduction:**

Nerve growth factor (NGF) has an important role in the generation of discogenic pain. We hypothesized that annular rupture is a trigger for discogenic pain through the action of NGF. In this study, the protein levels of NGF in discs from patients with disc herniation were examined and compared with those from discs of patients with other lumbar degenerative disc diseases.

**Methods:**

Patients (*n* = 55) with lumbar degenerative disc disease treated by surgery were included. Nucleus pulposus tissue (or herniated disc tissue) was surgically removed and homogenized; protein levels were quantified using an enzyme-linked immunosorbent assay (ELISA) for NGF. Levels of NGF in the discs were compared between 1) patients with herniated discs (herniated group) and those with other lumbar degenerative disc diseases (non-herniated group), and 2) low-grade and high-grade degenerated discs. Patient’s symptoms were assessed using a visual analog scale (VAS) and the Oswestry disability index (ODI); the influence of NGF levels on pre- and post-operative symptoms was examined.

**Results:**

Mean levels of NGF in discs of patients were significantly higher in herniated discs (83.4 pg/mg total protein) than those in non-herniated discs (68.4 pg/mg).

No significant differences in levels of NGF were found between low-grade and high-grade degenerated discs. Multivariate analysis, adjusted for age and sex, also showed significant correlation between the presence of disc herniation and NGF levels, though no significant correlation was found between disc degeneration and NGF levels. In both herniated and non-herniated groups, pre-operative symptoms were not related to NGF levels. In the herniated group, post-operative lower extremity pain and low back pain (LBP) in motion were greater in patients with low levels of NGF; no significant differences were found in the non-herniated group.

**Conclusions:**

This study reports that NGF increased in herniated discs, and may play an important role in the generation of discogenic pain. Analysis of patient symptoms revealed that pre-operative NGF levels were related to post-operative residual lower extremity pain and LBP in motion. The results suggest that NGF in the disc is related to pain generation, however, the impact of NGF on generation of LBP varies in individual patients.

**Electronic supplementary material:**

The online version of this article (doi:10.1186/ar4674) contains supplementary material, which is available to authorized users.

## Introduction

Despite its clinical importance, the pathogenesis of discogenic pain is poorly understood. Disc degeneration is thought to be one of the causes of chronic discogenic low back pain (LBP) [1]. However, the development of magnetic resonance imaging (MRI) has revealed that disc degeneration is commonly observed in patients without LBP, suggesting that the correlation between disc degeneration and pain is not clear [2–4].

It has been reported that intradiscal injections of local anesthetics and steroids eliminate acute LBP in patients with a radial annular tear; this suggests an annular rupture is one of the causes of acute LBP [5]. Acute discogenic LBP frequently disappears within one to two weeks. One possible explanation for the favorable clinical course is that an annular rupture usually heals spontaneously. Clinically, chronic discogenic pain is often preceded by one or more attacks of acute discogenic LBP [6]. These observations raise the possibility that acute LBP can be a trigger for development of chronic discogenic LBP.

Generally, the lumbar intervertebral disc has a high threshold to mechanical stimuli [7] and lacks sensory nerve fibers in the inner layer of the discs [8, 9], suggesting the lumbar disc is relatively insensitive to nociceptive stimuli under normal conditions. Previous studies using animal models show that annular rupture promotes (i) nerve ingrowth [10–12], (ii) an increase of inflammatory mediators [13, 14], and (iii) sensitization of nerve fibers in the disc [15], all of which are thought to cause chronic discogenic LBP in the human [9, 16–18]. These results support the hypothesis that annular rupture can be a trigger initiating development of chronic discogenic LBP.

Our previous studies show that disc-innervating neurons have extremely high sensitivity to nerve growth factor (NGF) [19, 20]. NGF has sensitizing and neurotrophic effects on the sensory nervous system, and has an important role in the generation of inflammatory pain states [21–23]. Therefore, we propose that NGF may act as a key factor in the generation of discogenic pain [19, 24].

Abe *et al*. reported that nucleus pulposus cells isolated from human discs express and synthesize NGF [25]. Freemont *et al*. revealed that NGF is present in painful, but not in asymptomatic, human discs [26]. Purmessur *et al*. demonstrated the localized expression of NGF by the fibroblast-like cells of human discs by immunohistochemistry [27]. Richardson *et al*. found increased expression of NGF mRNA in human discs from patients with chronic LBP, using quantitative real-time polymerase chain reaction [28]. From these observations, it is known that NGF is synthesized in the disc and may be related to the difference between asymptomatic and painful discs.

Therefore, we hypothesize that NGF is upregulated following annular rupture and may induce pathological changes, such as nerve ingrowth and sensitization, in the disc. If the upregulation persists, the disc becomes a source of chronic discogenic pain. To test this hypothesis, we first examined protein levels of NGF in discs from patients with disc herniation, and compared these levels with those in discs from patients with other lumbar degenerative diseases without disc herniation. Then we assessed the correlation between protein levels of NGF and disc degeneration. Finally, the correlation between protein levels of NGF and patient low back and lower extremity symptoms were examined.

## Methods

Fifty-five patients with lumbar degenerative disease, who received lumbar surgery in which a lumbar intervertebral disc was resected, were included in this study. Surgery for lumbar degenerative disease is routine clinical practice in our hospital. We surgically obtained 59 discs from patients with disc herniation (29 discs from 29 patients), or patients with other degenerated disc diseases (30 discs from 26 patients), such as spondylolisthesis, spinal canal stenosis, and lumbar degenerative scoliosis, and these were stored at -20°C. All the resected discs were evaluated using MRI; the Pfirmann grading was used to score disc degeneration from grade 1 (non-degenerated disc) to grade 5 (severely degenerated disc) [29]. Written informed consent was obtained prior to surgery. The study protocol was approved by the institutional ethics committee of Toho University Sakura Medical Center (number 2012-072).

### Evaluation of clinical symptoms in patients

Patient symptoms were assessed by visual analog scale (VAS) and Oswestry disability index (ODI). Pre-operative VAS scores (0 to 100 mm, for LBP, lower-extremity pain, and lower-extremity numbness) and the ODI were evaluated before surgery. In addition, our originally developed detailed VAS scoring system for LBP in motion, standing, and sitting [30] was used for a detailed evaluation of LBP. Postoperative data were prospectively acquired at one year following surgery.

### Measurement of NGF in the surgical samples

Nucleus pulposus tissue (or herniated disc tissue) was surgically removed and homogenized. Total protein was extracted with a Qproteome mammalian protein prep kit in accordance with the instructions of the manufacturer (Qiagen Inc., Valencia, CA, USA). Protein levels were quantified using a commercially available ELISA for beta-NGF (DY256, R&D Systems, Minneapolis, MN, USA). All samples were measured in duplicate, and the mean of the duplicate levels was used for statistical analysis. For each sample, data from the ELISA were normalized by the total protein concentration measured using the bicinchoninic acid (BCA) protein assay method (Pierce, Rockford, IL, USA).

### Evaluation of disc NGF levels and clinical data

Levels of NGF in the discs were compared between herniated discs (herniated group) and discs with other lumbar degenerative disc diseases (non-herniated group). In each group, discs were divided into two subgroups by the degree of disc degeneration: Pfirmann grades 2 to 3 (low-grade degeneration) versus Pfirmann grades 4 to 5 (high-grade) in the herniated group and Pfirmann grades 3 to 4 (low-grade degeneration) versus Pfirmann grade 5 (high-grade) in the non-herniated group. In each group, NGF levels in the discs were then compared between discs with low-grade and high-grade degeneration.

To investigate the relative influence of disc herniation, age, sex, and disc degeneration, the correlation of NGF levels with the presence of disc herniation, age, sex, and degree of disc degeneration was assessed. For comparison of patient symptoms, only data from patients with a single-level lumbar disc disorder without pathological changes in the other levels were used (herniated group: n = 24; non-herniated group: n = 12). The patients in each group were divided into two subgroups (low-NGF group and high-NGF group) according to NGF levels divided by median values, and the pre-operative VAS score, ODI and detailed VAS scores were compared between these two subgroups.

For the comparison of postoperative symptoms also, only data from patients with a single-level lumbar disc disorder were used. Patients who had factors affecting surgical results, such as post-surgical complication and recurrence of disc herniation, were excluded. In the herniated group, most patients were treated by decompression surgery without fusion, although some patients were treated by lumbar interbody fusion. However, only patients treated by decompression surgery were included for the analysis of postoperative data (n = 17). In the non-herniated group, all patients were treated by fusion surgery and were included for the analysis of postoperative data.

### Statistical analysis

For statistical analyses we used the unpaired *t*-test for NGF levels, age, and VAS scores, the Mann-Whitney *U-*test for ODI, and the chi-square test for sex and disc degeneration. Pearson’s correlation coefficient was used to analyze the correlation of NGF with age, and Spearman’s rank correlation coefficient was used to analyze the correlation of NGF with disc herniation, sex, and disc degeneration. Multiple linear regression analysis was used to investigate the relative influences of disc herniation and disc degeneration on NGF level in the discs, after adjusting for age and sex. Probability values <0.05 were considered significant. Values are expressed as the mean ± SD.

## Results

### Comparison of NGF levels between herniated discs and non-herniated discs

**Figure 1:**
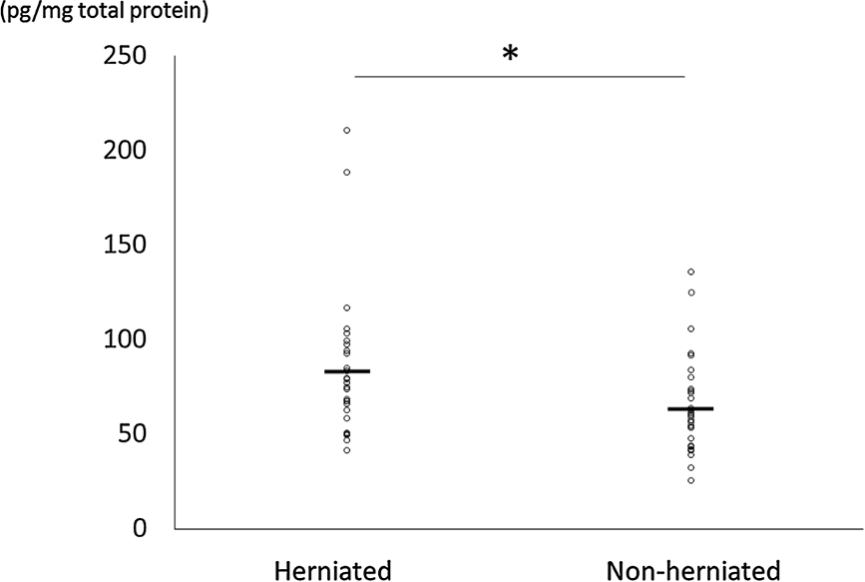
**Protein levels of nerve growth factor (NGF) measured using ELISA in herniated and non-herniated discs.** Data from the ELISA were normalized by total protein concentrations (pg/mg total protein). Mean values are indicated by short horizontal lines. The NGF level was significantly higher in herniated discs. *P <0.05.

Mean levels of NGF in discs of patients were 83.4 ± 37.7 pg/mg total protein in the herniated group and 64.8 ± 25.9 pg/mg total protein in the non-herniated group. The herniated group showed significantly higher levels of NGF (Figure 1; *P* = 0.031).

The pre-operative characteristics of the patients in each group are shown in Additional file 1: Table S1. The mean patient age was significantly younger, and the grade of disc degeneration was significantly lower in the herniated group (Additional file 1: Table S1; *P* <0.01).

### Comparison of NGF levels between discs with low-and high-grade degeneration

**Figure 2:**
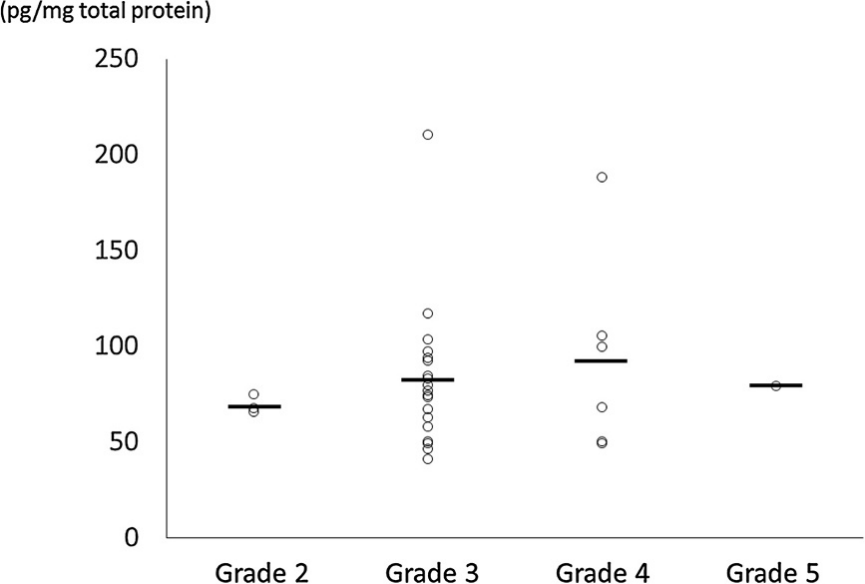
**Protein levels of nerve growth factor (NGF, pg/mg total protein) measured using ELISA in herniated discs.** Mean values are indicated by short horizontal lines. No significant correlation was found between NGF levels and the grade of disc degeneration (Pfirmann grade).

**Figure 3:**
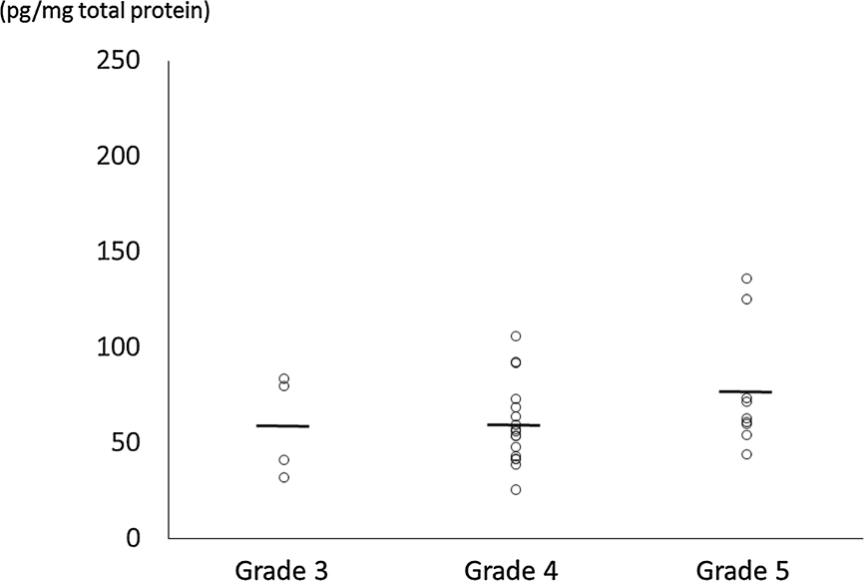
**Protein levels of nerve growth factor (NGF, pg/mg total protein) measured using ELISA in non-herniated discs.** Mean values are indicated by short horizontal lines. No significant correlation was found between NGF levels and the grade of disc degeneration (Pfirmann grade).

In the herniated group, mean disc levels of NGF were 80.8 ± 34.8 pg/mg total protein in the discs with low-grade degeneration (Pfirmann grade 2 to 3, n = 22), and 91.8 ± 47.9 pg/mg total protein in the discs with high grade degeneration (Pfirmann grade 4 to 5, n = 7). Although highly degenerated discs in the herniated group had higher levels of NGF, no significant differences in levels of NGF were found (*P* = 0.51; Figure 2). In the non-herniated group, mean levels of NGF were 59.7 ± 21.6 pg/mg total protein in the discs with low-grade degeneration (Pfirmann grade 3 to 4, n = 21), and 76.7 ± 32.0 pg/mg total protein in the discs with high-grade degeneration (Pfirmann grade 5, n = 9). Similar to the herniated disc group, no significant differences in levels of NGF were found between non-herniated discs with low-grade degeneration and discs with high-grade degeneration (*P* = 0.10; Figure 3).

### Correlation between NGF level and the presence of disc herniation, age, sex, and the degree of disc degeneration

Pearson’s correlation analysis showed no significant correlation between level of NGF and age. Spearman’s rank correlation analysis showed significant correlation between NGF and the presence of disc herniation (Spearman’s correlation coefficient = - 0.311, *P* <0.05), but no significant correlation between NGF and either sex or disc degeneration was found.

A multivariate analysis was performed, adjusted for age and sex, with the presence of disc herniation and disc degeneration as independent variables, and the NGF level as the dependent variable. The results showed significant correlation between the presence of disc herniation and NGF levels (Additional file 1: Table S2; *P* = 0.019), however, no significant correlation was found between disc degeneration and NGF levels (Additional file 1: Table S2; *P* = 0.224).

### Effects of disc levels of NGF on pre-operative and postoperative symptoms

In the herniated group, pre-operative data obtained from 24 patients were compared between patients with low-level NGF (n = 12) and high-level NGF (n = 12); no significant difference was observed between the two groups in any of the VAS scores, the ODI, or the detailed VAS scores.

A year after surgery, postoperative data for the VAS score, the ODI and the detailed VAS scores were collected. In the herniated group, as already mentioned, only patients treated by decompression surgery were included for the analysis of postoperative data (n = 17) of the 17 patients 14 were followed up at 1 year after surgery, and in the remaining 3 patients, postoperative data collected at 6 months after the surgery were used. All of the VAS scores, the ODI and the detailed VAS scores showed significant improvement after surgery. In the low-level NGF group, the postoperative VAS for LBP showed a non-significant tendency toward greater residual LBP than in the high-level NGF group (*P* = 0.064, Additional file 1: Table S3). In the low-level NGF group, the postoperative VAS scores for lower extremity pain and LBP in motion were significantly greater compared to the high-NGF group (Additional file 1: Table S3).

In the non-herniated group, pre-operative data obtained from 12 patients were compared between patients in the low-level NGF group (n = 6) and the high-level NGF group (n = 6); again, no significant differences were observed between the two groups in any of the VAS scores, the ODI or the detailed VAS scores (Additional file 1: Table S4). All 12 patients were treated by fusion surgery and evaluated at 1 year after surgery; none of the postoperative data showed a significant difference between the two groups (Additional file 1: Table S4).

## Discussion

Previous studies using immunohistochemistry show the expression of NGF in microvascular blood vessels [26], and chondrocyte-like cells [27, 31] in human intervertebral discs. The presence of NGF-sensitive sensory fibers in human symptomatic discs has also been reported [20, 26]. These findings suggest that NGF may regulate nerve ingrowth into degenerated discs. Considering that NGF has the potential to sensitize sensory neurons [32, 33], as well as to promote axonal growth [34, 35], NGF expressed in human discs may initiate the development of discogenic pain.

This study reports that herniated intervertebral discs show increased protein levels of NGF, which may lead to pathological nerve ingrowth into the disc [35, 36]. It has been suggested that nerve ingrowth may be triggered by annular rupture [10, 11, 16]. It is believed that nerve fibers hardly extend into the normal disc without annular rupture or disc degeneration because of the tight collagen network of the uninjured annulus and the presence of disc aggrecan, which has an inhibiting effect on extending nerve fibers [37]. However, destruction of the annulus and increased NGF following disc herniation (annular rupture) may give sensory nerve fibers an opportunity to extend further into the disc.

Lee *et al*. reported the results of their experiment that determined the expression of NGF in the disc was significantly higher in degenerative disc disease than in disc herniation [38]. They examined the expression of NGF using western blot analysis, whereas we used ELISA. The mean age of the 10 patients enrolled in their study as the degenerated disc disease group was 53.4 ± 8.8 years, whereas the mean age of the non-herniated group in our study was 72.0 ± 10.0 years. Moreover, all 10 patients in their study were female, whereas 9 of the 30 patients in the non-herniated group of our study were male. Differences in timing of surgery and indications for surgery might also explain the different results. In our study, we evaluated the correlation between NGF levels and the presence of disc herniation after adjusting for age, sex and the degree of disc degeneration, and this analysis showed significant correlation. From these observations, we believe our results are more reliable and conclude that in most cases NGF increased in the herniated discs. However, as shown in Figure 1, some of the herniated discs had low NGF levels compared with non-herniated discs. There is a possibility that other factors influencing NGF level in the disc will be found by future studies.

The present study has several limitations. First, the cells expressing NGF were not identified. Previous studies have reported that various cells, such as chondrocyte-like cells [27, 31], blood vessels [26], and inflammatory cells [39], secrete NGF. The most important finding in our study is the fact that protein levels of NGF were increased in ruptured discs, because NGF may act as a pain generator regardless of which cells express NGF in the disc. Second, annular rupture frequently occurs during natural degenerative processes [40, 41], suggesting discs from the non-herniated group may also have annular rupture. Because of this issue, data interpretation is complicated. However, in our study, most annular ruptures in non-herniated discs may have been in the chronic phase, whereas annular ruptures in herniated discs were mostly in acute or sub-acute phases. Moreover, significant differences in NGF levels were found between herniated and non-herniated discs, though there is a possibility that a certain number of non-herniated discs may have acute or sub-acute annular ruptures. From these observations, we believe the second limitation is not a critical issue influencing our conclusions.

The correlation between NGF expression and disc degeneration is not clear cut. Purmessur *et al*., using immunohistochemistry, showed no significant difference in the expression of NGF between non-degenerated and degenerated discs [27]. Richardson *et al*., using quantitative real-time polymerase chain reaction, described the increased expression of NGF mRNA in the symptomatic degenerated human intervertebral disc [28]. In our study, no significant difference in NGF levels was detected between low-grade and high-grade degenerated discs, however, for the non-herniated group the *P-*value (*P* = 0.10) was relatively low for analysis of NGF levels and disc degeneration. Therefore, we further evaluated the correlation between NGF levels and disc degeneration after adjusting for age and sex, and there was no significant correlation (*P* = 0.224). From these results we could at least exclude strong correlation between NGF levels and disc degeneration. However, correlation between disc degeneration and NGF levels is still open to question and further investigation is needed to reach a conclusion.

The strong point of the present study is that any correlation between NGF and clinical symptoms, both pre-operative and postoperative, was thoroughly analyzed. No significant differences in pre-operative symptoms were found between high-level NGF and low-level NGF groups in either the herniated or non-herniated groups. However, in the herniated group, post-operative LBP was lower in the patients with high levels of NGF in the disc. Interestingly, our original detailed VAS scoring system for LBP in motion, standing and sitting successfully detected a significant difference in postoperative LBP in motion between the high-NGF group and the low-NGF group. These results lead us to conclude that NGF produced in the herniated discs may at least to some degree be related to the LBP of these patients.

The high-level NGF group also had more improvement in lower extremity pain. The cause of lower extremity pain in disc herniation is generally recognized to be a result of mechanical compression, as well as inflammation [42, 43]. It is also generally known that the herniated disc contains various inflammatory mediators, such as IL-1β, IL-6, and TNF-α [44–46]. As previously mentioned, NGF plays an important role in the generation of inflammatory pain, and is regulated by other inflammatory mediators [21–23]. Thus, a possible explanation for these results is that inflammatory pain, in which NGF is involved, is more intensely related to pre-operative lower extremity pain in the high-level NGF group. In such patients, the inflammatory pain, which may be mainly related to LBP in motion and lower extremity pain, may dramatically improve after removal of the herniated tissue containing NGF.

In 2010, Lane *et al*. reported the results of a randomized trial of 450 patients with osteoarthritis of the knee; the authors concluded that anti-NGF treatment produced significant reduction in pain [47]. A more recent clinical study demonstrated the favorable effects of anti-NGF treatment on chronic low back pain patients [48]. Kumar *et al*. suggested the existence of responders and non responders to anti-NGF treatment, and that the difference in response may be due to pretreatment NGF levels [49]. From these observations, we propose that our results may explain the different response to anti-NGF therapy, in that the degree of functional involvement of NGF in pain generation varied among individuals.

In the non-herniated group, no significant difference between the high-level and low-level NGF groups was found in any of the postoperative symptom data. Because these patients were treated by fusion surgery and mechanical stress to the symptomatic disc was completely eliminated, we believe that the pain derived from the disc was completely resolved irrespective of NGF levels. Thus, in the non-herniated group, it seems reasonable that postoperative symptoms in the high-level NGF group were similar to those of the low-level NGF group. This suggests that postoperative pain after fusion surgery may be due to factors other than surgery to the disc.

Our study found no significant differences in pre-operative LBP between the herniated and non-herniated groups, and does not prove the hypothesis that NGF upregulation persists in the disc to cause chronic discogenic LBP. However, a limitation of our study is that the subjects’ back pain was not necessarily caused by disc herniation or disc degeneration. Future studies in which inclusion criterion are strictly limited to patients with discogenic pain are expected to elucidate the correlation between NGF and back pain in these patients.

## Conclusions

Because numerous studies support the important role of NGF in the generation of discogenic pain, the results of the present study raise the possibility that NGF produced following annular rupture may, at least in some cases, initiate the development of discogenic back pain. We have to keep in mind that most annular ruptures do not necessarily cause chronic discogenic LBP [40, 41]. However, chronic discogenic LBP develops when pathological changes, such as nerve ingrowth and sensitization, are induced by NGF produced following annular rupture. Our results showing that pre-operative disc protein levels of NGF affect postoperative LBP and lower extremity pain, suggest there is some degree of impact of NGF on generation of LBP and lower extremity pain that varies among individual patients.

## Electronic supplementary material

Additional file 1: Table S1: Pre-operative characteristics of patients in herniated group and non-herniated group. **Table S2.** Correlation between the measured parameters and the level of nerve growth factor in lumbar intervertebral discs. **Table S3.** Comparisons of pre- and post-operative visual analog scale (VAS) scores, Oswestry disability index (ODI), and detailed VAS scores for back pain (in motion, standing, and sitting) in patients with disc herniation, between low-level and high-level disc nerve growth factor (NGF) groups. **Table S4.** Comparisons of pre- and post-operative visual analog scale (VAS) scores, Oswestry disability index (ODI), and detailed VAS scores for back pain (in motion, standing, and sitting) in non-herniated disc patients, between low-level and high-level disc nerve growth factor (NGF) groups. (DOC 68 KB)

## Abbreviations

ELISA: enzyme-linked immunosorbent assay
IL: interleukin
LBP: low back pain
MRI: magnetic resonance imaging
NGF: nerve growth factor
ODI: Oswestry disability index
TNF: tumor necrosis factor
VAS: visual analog scale.
