# Supplementary material for: Increase of nerve growth factor levels in the human herniated intervertebral disc: can annular rupture trigger discogenic back pain?
Source: Arthritis Res Ther. 2014 Jul 28;16(4):R159. doi: 10.1186/ar4674 (PMC4261264; doi:10.1186/ar4674)
Supplement: Supplementary file 1 — Additional file 1: Table S1: Pre-operative characteristics of patients in herniated group and non-herniated group. Table S2. Correlation between the measured parameters and the level of nerve growth factor in lumbar intervertebral discs. Table S3. Comparisons of pre- and post-operative visual analog scale (VAS) scores, Oswestry disability index (ODI), and detailed VAS scores for back pain (in motion, standing, and sitting) in patients with disc herniation, between low-level and high-level disc nerve growth factor (NGF) groups. Table S4. Comparisons of pre- and post-operative visual analog scale (VAS) scores, Oswestry disability index (ODI), and detailed VAS scores for back pain (in motion, standing, and sitting) in non-herniated disc patients, between low-level and high-level disc nerve growth factor (NGF) groups. (DOC 68 KB) [file 13075_2014_4245_MOESM1_ESM.doc]

Table 1.

Pre-operative characteristics of patients in herniated group and non-herniated group.

|  |  | Herniated  (n=29) | Non-herniated  (n=30) | *p* value |
| --- | --- | --- | --- | --- |
| Age | (years) | 44.9 ± 15.9 | 72.0 ± 10.0 | *p* < 0.01 |
| Sex | (M/F) | 16 / 13 | 9 / 21 | *p* = 0.09 |
| Disc degeneration | Pfirmann  Grade 2/3/4/5 | 3 / 19 / 6 / 1 | 0 / 4 / 17 / 9 | *p* < 0.01 |

Table 2.

Correlation between the measured parameters and the level of nerve growth factor in lumbar intervertebral discs.

| Independent valuable | Regression coefficient | Standardized regression coefficient | t value | *p* value |
| --- | --- | --- | --- | --- |
| Presence of disc herniation | -30.840 | -0.467 | 2.413 | *p* = 0.019 |
| Disc degeneration | 8.776 | 0.094 | 1.230 | *p* = 0.224 |

Table 3.

Comparisons of pre- and post-operative visual analogue scale (VAS) scores, Oswestry disability index (ODI), and detailed VAS scores for back pain (in motion, standing, and sitting) in patients with disc herniation, between low-level and high-level disc nerve growth factor (NGF) groups.

|  |  | Low-level NGF (n=8) | High-level NGF (n=9) | *p* value |
| --- | --- | --- | --- | --- |
| Age | (years) | 38.9± 13.6 | 38.8 ± 11.1 | NS |
| Sex | (M/F) | 4 / 4 | 5 / 4 | NS |
| NGF levels | pg/mg total protein | 66.4 ± 9.1 | 118.3 ± 47.9 | *p* = 0.009 |
| Disc degeneration | Pfirmann  Grade 2/3/4 | 2 / 6 / 0 | 0 / 6 / 3 | NS |
| Low back pain (LBP) | Pre-op | 53.9 ± 36.0 | 53.9 ± 31.9 | NS |
|  | Post-op | 12.5 ± 11.4 | 4.1 ± 5.2 | *p* = 0.064 |
| Lower extremity pain | Pre-op | 87.5 ± 10.6 | 71.8 ± 37.5 | NS |
|  | Post-op | 12.9 ± 8.2 | 3.1 ± 4.9 | *p* = 0.009 |
| Lower extremity numbness | Pre-op | 55.0 ± 38.7 | 69.8 ± 32.4 | NS |
|  | Post-op | 10.6 ± 11.6 | 8.7 ± 14.2 | NS |
| ODI | Pre-op | 50.3 ± 13.2 | 48.4 ± 19.8 | NS |
|  | Post-op | 7.5 ± 5.4 | 8.5 ± 9.8 | NS |
| LBP in motion | Pre-op | 71.5 ± 29.7 | 52.3 ± 36.9 | NS |
|  | Post-op | 8.0 ± 7.4 | 1.3 ± 3.3 | *p* = 0.029 |
| LBP in standing | Pre-op | 66.1 ± 34.4 | 46.4 ± 40.4 | NS |
|  | Post-op | 10.6 ± 12.6 | 2.9 ± 3.9 | NS |
| LBP in sitting | Pre-op | 65.6 ± 30.1 | 47.9 ± 37.6 | NS |
|  | Post-op | 9.4 ± 8.1 | 7.7 ± 13.8 | NS |

Values are expressed as the mean ±standard deviation. NS is non-significant at p>0.05

Table 4.

Comparisons of pre- and post-operative visual analogue scale (VAS) scores, Oswestry disability index (ODI), and detailed VAS scores for back pain (in motion, standing, and sitting) in non-herniated disc patients, between low-level and high-level disc nerve growth factor (NGF) groups.

|  |  | Low-level NGF (n=6) | High-level NGF (n=6) | *p* value |
| --- | --- | --- | --- | --- |
| Age | (years) | 65.7 ± 6.1 | 72.2 ± 14.8 | NS |
| Sex | (M/F) | 4 / 2 | 3 / 3 | NS |
| NGF levels | pg/mg total protein | 40.3 ± 10.8 | 84.4 ± 16.4 | *p* = 0.0003 |
| Disc degeneration | Pfirmann  Grade 2/3/4 | 2 / 4 / 0 | 1 / 3 / 2 | NS |
| Low back pain (LBP) | Pre-op | 51.8 ± 32.5 | 51.2 ± 17.8 | NS |
|  | Post-op | 16.3 ± 25.8 | 22.7 ± 20.8 | NS |
| Lower extremity pain | Pre-op | 67.7 ± 21.2 | 62.8 ± 17.6 | NS |
|  | Post-op | 23.8 ± 30.0 | 13.7 ± 21.9 | NS |
| Lower extremity numbness | Pre-op | 76.3 ± 11.1 | 68.7 ± 11.5 | NS |
|  | Post-op | 21.0 ± 32.1 | 21.3 ± 21.6 | NS |
| ODI | Pre-op | 37.2 ± 10.1 | 40.6 ± 13.1 | NS |
|  | Post-op | 15.6 ± 14.8 | 27.9 ± 25.6 | NS |
| LBP in motion | Pre-op | 41.7 ± 28.3 | 42.8 ± 20.4 | NS |
|  | Post-op | 17.2 ± 27.8 | 29.2 ± 33.5 | NS |
| LBP in standing | Pre-op | 62.2 ± 30.5 | 58.0 ± 24.7 | NS |
|  | Post-op | 8.0 ± 6.9 | 23.3 ± 22.5 | NS |
| LBP in sitting | Pre-op | 33.7 ± 20.0 | 30.7 ± 19.0 | NS |
|  | Post-op | 4.0 ± 6.3 | 16.7 ± 27.3 | NS |

Values are expressed as the mean ±standard deviation. NS is non-significant at p>0.05
